# Case Report: Exercise-induced normalization of chronic left bundle branch block: a rare recovery-phase phenomenon

**DOI:** 10.3389/fphys.2025.1745858

**Published:** 2026-01-08

**Authors:** Horesh Dor-haim, Ilia Davarashvili

**Affiliations:** 1 Malcha Heart Institute, Jerusalem, Israel; 2 O2 Health Promotion and Sports Medicine Department, The Howard and Mary Edith Cosell Association for Physical Education, The Safra Sports Center, Hebrew University, Jerusalem, Israel; 3 Leumit Healthcare Services, Internal Medicine and Cardiology, Hebrew University, Jerusalem, Israel

**Keywords:** coronary artery disease, electrophysiology, left bundle branch block, stress echocardiogram, transient conduction abnormalities

## Abstract

**Background:**

Transient left bundle branch block (LBBB) is associated with various cardiac and systemic conditions, including exercise-induced ischemia. However, normalization of baseline LBBB following exertion is a rare and poorly understood phenomenon.

**Case Summary:**

We report the case of an 83-year-old male with longstanding coronary artery disease and baseline LBBB who underwent a routine stress echocardiogram. During the recovery phase, ECG monitoring revealed normalization of LBBB with first-degree AV block. Concurrent stress echocardiography showed improved segmental and global ventricular function.

**Conclusion:**

This case highlights a rare instance of post-exercise resolution of LBBB and suggests a possible link between exercise-induced autonomic modulation and transient improvement in conduction. Further research is warranted to explore the prognostic significance of this finding in patients with structural heart disease.

## Introduction

Left bundle branch block (LBBB) is a common conduction abnormality defined by delayed activation of the left ventricle due to impaired transmission through the left bundle of the His–Purkinje system. Its presence on electrocardiogram (ECG) often signifies underlying structural heart disease, including ischemic heart disease, dilated cardiomyopathy, or degeneration of the conduction system. In many cases, LBBB is persistent and static. However, transient bundle branch block reflects dynamic alterations in intraventricular conduction rather than irreversible anatomical damage, as originally described by Bauer (Bauer, 1964). Transient or rate-dependent LBBB has been documented under various clinical contexts such as exertion, ischemia, myocarditis, trauma, and iatrogenic procedures, including valve replacement or device implantation (Issa et al., 2012).

Transient LBBB during exertion, specifically exercise-induced LBBB (EI-LBBB), is a rare manifestation occurring in approximately 0.38%–0.5% of individuals undergoing treadmill stress testing, typically at heart rates between 120 and 140 beats per minute (Stein et al., 2011). Studies have demonstrated that EI-LBBB is associated with adverse outcomes, including increased long-term mortality and higher rates of major adverse cardiac events compared with matched controls (Grady et al., 1998). Most reports describe LBBB emerging during exercise due to rate-related or ischemia-related conduction delay (Issa et al., 2012; Stein et al., 2011; Oladunjoye et al., 2019). However, normalization of pre-existing baseline LBBB during or after exercise—particularly during the recovery phase—has been only sparsely documented.

Clinically, LBBB is traditionally regarded as a fixed manifestation of advanced conduction system disease with important diagnostic and prognostic implications. LBBB induces interventricular and intraventricular desynchrony, impairs left ventricular systolic and diastolic performance, and creates characteristic septal motion and perfusion abnormalities that complicate interpretation of stress imaging studies (Littmann and Symanski, 2000; Xiao and Gibson, 1994; Canna et al., 1992). Consequently, recovery-phase normalization of baseline LBBB is an electrophysiological rare phenomenon which may reflect latent conduction reserve within the His–Purkinje system. Recognition of this phenomenon has potential implications for interpretation of stress testing, understanding conduction system plasticity, and risk stratification in patients with coronary artery disease (CAD).

## Case report

We report the case of an 83-year-old male with a long-standing history of coronary artery disease (CAD) and chronic complete left bundle branch block, documented on serial baseline electrocardiograms. His medical history was significant for hypertension, hyperlipidemia, chronic obstructive pulmonary disease, peripheral artery disease, paroxysmal atrial fibrillation on long-term anticoagulation, and prior tobacco use.

At the age of 59, the patient underwent coronary artery bypass grafting, with the left internal mammary artery sequentially grafted to the left anterior descending artery and first diagonal branch, and the right internal mammary artery grafted to the first obtuse marginal branch. Over subsequent decades, he experienced multiple episodes of acute coronary syndrome requiring repeat coronary angiography and multiple percutaneous coronary interventions to the LAD, left circumflex artery, and right coronary artery, including several procedures for in-stent restenosis, predominantly in the LAD territory.

Baseline transthoracic echocardiography performed 1 year prior to the current evaluation demonstrated a mildly reduced left ventricular ejection fraction of approximately 45%, with mid- and apical septal akinesis and global hypokinesis. There was mild-to-moderate biatrial enlargement, mild mitral and tricuspid regurgitation, and normal pulmonary artery pressures, with preserved right ventricular size and function. A single-photon emission computed tomography (SPECT) myocardial perfusion study demonstrated a permanent perfusion defect in the septum, attributed to a prior myocardial infarction in conjunction with chronic LBBB. Additionally, a small non-reversible defect was noted in the RCA territory, suggestive of a previous infarction.

Chronic medications included omeprazole 20 mg daily, apixaban 5 mg twice daily, ramipril 2.5 mg twice daily, rosuvastatin 20 mg daily and ezetimibe 10 mg daily. Blood pressure and metabolic parameters were well controlled.

**FIGURE 1:**
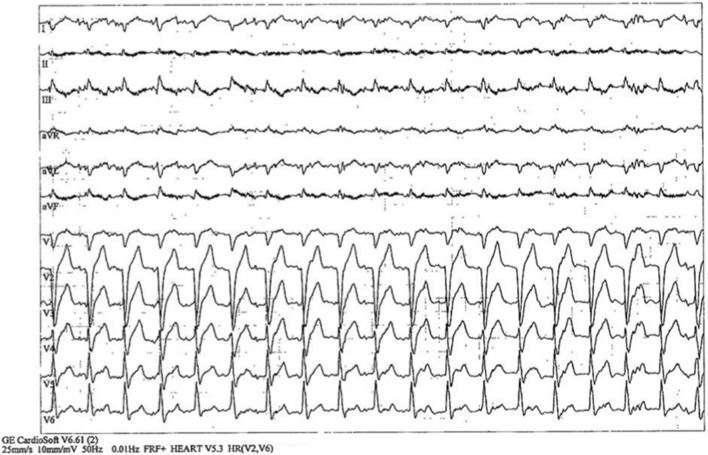
Twelve-lead electrocardiogram during peak exercise demonstrating sinus rhythm with complete left bundle branch block and wide QRS complexes.

As part of routine follow-up, the patient underwent exercise stress testing combined with echocardiography. Resting ECG showed sinus rhythm (88 bpm) with complete LBBB, which persisted throughout all stages of exercise, with appropriate chronotropic response (Figure 1). The test was terminated at 97% of the age-predicted maximal heart rate (peak heart rate 133 bpm; stage II; 5:15 min). Systolic blood pressure increased from 125/75 mmHg at rest to 160/90 mmHg during exertion.

**FIGURE 2:**
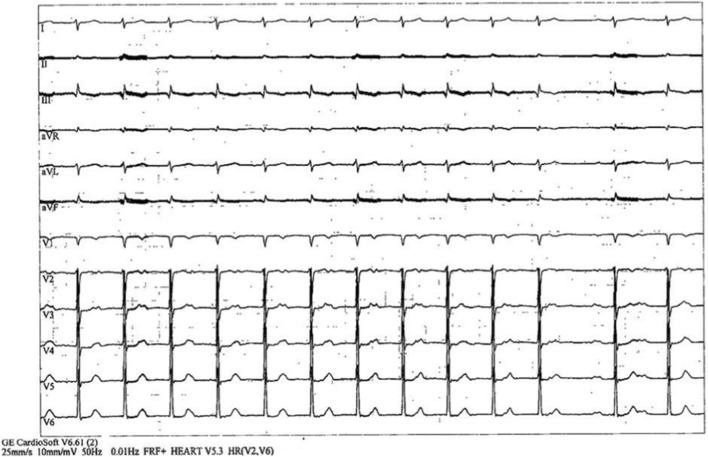
Twelve-lead electrocardiogram during the recovery phase demonstrating normalization of QRS duration with first-degree atrioventricular block.

Unexpectedly, during the third minute of recovery, ECG monitoring demonstrated a transition from a wide QRS complex consistent with LBBB to a narrow QRS complex with first-degree atrioventricular block (HR: 82 bpm; PR interval 220 m), which persisted throughout the remainder (8 min) of the recovery phase (Figure 2).

Stress echocardiography demonstrated a baseline LVEF of approximately 45%, with mid- and apical septal akinesis (septal wall motion scoring-4). At peak exercise and persisting into recovery, there was qualitative and improvement in septal thickening (septal wall motion scoring 2-3) and left ventricular global systolic performance increased (LVEF post-exercise increased to approximately to 55% corresponding with normalization of intraventricular conduction and overall synchrony. No inducible ischemia or new wall motion abnormalities were observed and the patient remained hemodynamically stable and asymptomatic throughout.

## Discussion

The mechanisms underlying transient or reversible bundle branch block are multifactorial and may reflect dynamic interactions between heart rate, refractoriness of the His–Purkinje system, autonomic modulation, transient ischemic and myocardial substrate changes.

The classical electrophysiological descriptions distinguish rate-dependent phase 3 block—where impulses encounter tissue in a relative refractory period at higher heart rates—from phase 4 (bradycardia-dependent) block, which occurs in diseased Purkinje fibers during prolonged diastolic intervals (Issa et al., 2012). These mechanisms explain the more common emergence of EI-LBBB during exertion.

One of the mechanisms relevant to the present case is the “linking phenomenon,” whereby persistent retrograde activation from the contralateral bundle maintains the affected bundle in a refractory state, preventing antegrade conduction despite normalization of triggering conditions (Erdogan and Altun, 2002; Caldwell et al., 2013). As heart rate decreases during recovery and retrograde penetration diminishes, conduction through the previously blocked left bundle may be restored, resulting in transient normalization of the QRS complex.

Autonomic modulation likely plays a pivotal role. Exercise is characterized by heightened sympathetic tone, whereas the recovery phase is marked by rapid parasympathetic reactivation. Stewart et al. demonstrated that post-exercise autonomic shifts are associated with measurable changes in cardiac electrical conduction and repolarization (Stewart et al., 2014). Support for exercise-induced modulation of autonomic mechanistic framework is found in animal and human studies. Aerobic exercise reduced ventricular arrhythmogenicity and increased intracardiac electrical stability, driven largely by autonomic remodeling (Dor-Haim et al., 2013). Improved vagal tone and spatial repolarization uniformity were crucial in promoting conduction stability. Chronic as well as acute exercise induce parasympathetic predominance and myocardial conduction synchrony (Dor-Haim et al., 2022).

In the present case, the emergence of first-degree atrioventricular block concurrent with normalization of intraventricular conduction strongly supports increased vagal tone during recovery. While parasympathetic activation slows AV nodal conduction, it may simultaneously stabilize distal His–Purkinje fiber excitability, facilitating recovery of conduction within a partially diseased left bundle.

Alternative explanations may consider ischemic-mediated improvement in conduction is less likely given the absence of angina, ischemic ECG changes, or inducible wall motion abnormalities (Canna et al., 1992). Furthermore, the persistence of LBBB throughout peak exercise argues against simple rate-related aberrancy as the primary mechanism. Instead, the findings are most consistent with dynamic conduction system reserve or “mobility,” wherein residual viable fibers transiently recover under favorable autonomic and metabolic conditions.

Multiple studies indicate that EI-LBBB is associated with a higher risk of adverse cardiac outcomes, including a 29% 5-year mortality rate and 19% incidence of major adverse cardiac events, compared to 25% and 10% in matched controls (Grady et al., 1998). LBBB, when present, alters left ventricular contraction by inducing dyssynchrony, impeding early septal activation, and disrupting mitral valve timing. These abnormalities lead to both systolic and diastolic dysfunction (Littmann and Symanski, 2000; Xiao and Gibson, 1994), which in patients with existing ischemic heart disease, can exacerbate heart failure and increase arrhythmic risk. LBBB induces interventricular and intraventricular dyssynchrony, resulting in inefficient ventricular contraction, abnormal septal motion, and impaired diastolic filling (Littmann and Symanski, 2000; Xiao and Gibson, 1994). In this case, normalization of intraventricular conduction during recovery coincided with improvement in regional septal thickening and global systolic performance, supporting the concept that restoration of electrical synchrony directly enhances mechanical efficiency. Experimental and clinical studies demonstrate that exercise training improves cardiac electrical stability and autonomic balance, reducing ventricular arrhythmogenicity and enhancing conduction uniformity (Dor-Haim et al., 2013; Dor-Haim et al., 2022).

This case highlights, LBBB resolution during recovery can lead to a transient restoration of synchronous ventricular activation. Stress echocardiography in our patient showed improved segmental wall motion and global function, suggesting that normalization of electrical conduction restores mechanical efficiency. This adds diagnostic value to recovery-phase ECG analysis, a phase often underappreciated in clinical protocols focused solely on exercise-induced ischemia.

Lastly, the transient improvements in both electrical conduction and myocardial wall motion observed during the post-exercise recovery phase in this case may indicate the presence of conduction reserve. These reversible electromechanical changes, unmasked by exercise testing, highlight the dynamic nature of cardiac conduction and contractile function. Future research focusing on such exercise-induced electromechanical responses may provide valuable insights into myocardial adaptability and conduction system integrity, potentially enhancing patient-specific risk stratification and informing therapeutic decision-making.

## Conclusion

This case highlights a rare but clinically meaningful phenomenon: recovery-phase normalization of chronic left bundle branch block following exercise stress testing. The observation suggests preserved conduction reserve within the diseased His–Purkinje system, likely unmasked by post-exercise autonomic rebalancing and dynamic refractory changes. Importantly, restoration of synchronous ventricular activation was accompanied by measurable improvement in left ventricular mechanical performance.

From a clinical standpoint, careful ECG evaluation during the recovery phase of stress testing—often underemphasized in routine protocols—may yield important diagnostic and prognostic insights. Recovery-phase normalization of LBBB may identify a subset of patients with CAD and conduction abnormalities who exhibit reversible electromechanical dysfunction and potentially distinct prognostic trajectories. Further investigation is warranted to determine the also the benefit of chronic exercise in LBBB and whether this phenomenon has implications for stress test interpretation, conduction system assessment, or therapeutic decision-making in patients with chronic LBBB.

## Data Availability

The original contributions presented in the study are included in the article/supplementary material, further inquiries can be directed to the corresponding author.
